# Hypodermic Use of Belladonna and Atropia in Cholera

**Published:** 1873-09-15

**Authors:** C. Truesdale

**Affiliations:** Rock Island


### HYPODERMIC USE OF BELLADONNA AND ATROPIA IN CHOLERA.

EDITORS MED. EXAMINER: *Dear Sirs*—I take the liberty of addressing you this note, for the purpose of inquiring whether you have had any experience in the hypodermic use of belladonna or atropia in the treatment of cholera morbus, cholera infantum, or Asiatic cholera; also, to detail briefly the history of a few cases, illustrating my experience with these remedies as above indicated.

July 25th, was called to visit Mrs. E., a German lady, 55 years old, who I found suffering from a severe attack of cholera morbus. Retching, vomiting and purging almost continuously, with severe cramps in legs and feet; in fact, was in incipient collapse: extremities cold and blue; pulse feeble and thready. I at once injected, hypodermically, one-fourth gr. acet. morph. over the stomach, and soon after five drops of the fluid ext. belladonna, in same manner, over the upper part of the spine. Vomiting, purging and cramps ceased immediately. For six hours no other medicines were given. Reaction was perfect within two hours. At the end of six hours purging manifested a disposition to return. I administered three drops of the belladonna as before, and following this recovery was rapid and complete.

July 30th, was called to see a child of Mr. S., two years old. Found it sick of cholera infantum; vomiting and purging almost constantly; eyes shrunken and glassy; body bathed in a cold perspiration; extremities cold and becoming dark; in short, it was a case that appeared hopeless. I at once injected over the upper part of the spine three drops of the fluid ext. belladonna, and waited the result. There was no more vomiting or purging; the pulse became fuller; the extreme restlessness ceased; and in half an hour the child was in a glow of heat from head to toes. Recovery was rapid and complete, without other medication.

Aug. 2d, was called to meet Mrs. S., a lady 35 years old, in consultation with Dr. Galt. I found her in what at that time seemed to be a hopelessly collapsed condition. Had been sick about 36 hours, and had manifested all the characteristic symptoms of Asiatic cholera. She had been in a collapsed condition for several hours; pulse at the wrist imperceptible; the extremities, of course, cold and blue, as usual in those cases. All the ordinary remedies had been given. The most powerful diffusible stimulants, such as camphor, ammonia, and sulph. ether, made no impression. I had related to Dr. Galt my experience with belladonna. We concluded it would be an excellent case in which to test its virtues. We secured a solution of atropia, of the strength of one gr. to an half oz. of water. Of this, we injected over the lower part of the spine ten drops, and waited half an hour to watch the result. The first noticeable effect was in the almost total relief of the restlessness; next was a change in the appearance of the skin from dark to scarlet; finally, the pulse began to gather strength. We waited an hour, and observing no marked change in the appearance of the pupils, concluded to repeat the injections. We injected two drops more over the deltoid muscle. From this on, reaction, although slow, gradually progressed, and became complete. Nothing else was used.

Although in this case one-twelfth of a gr. of atropia was used within an hour, no very marked impression was produced upon the pupils.

I have not used atropia or belladonna in a single case of cholera morbus where the relief has not been prompt and decided. I have not saved all the cases of cholera infantum in which the belladonna has been used, but have never failed in mitigating the symptoms.

I have a theory upon which this treatment is based, but have not time at present to give it in full.

I understand you are having the Asiatic cholera in Chicago; and I should like to have you, or some other members of the profession, test the virtues of belladonna in all the choleraic class of diseases.

Very truly,
C. TRUESDALE, M.D.

Rock Island, Aug. 25th, 1873.

